# Dr. George A. Edwards

**Published:** 1914-01

**Authors:** 


Dr. George A. Edwards, P. & S. (Columbia), 1877, died at his home in Syracuse in November, 1913.

